# Supplementary material for: Proprotein convertase activity regulates cumulus-oocyte-complex matrix integrity and cumulus cell migration during ovulation via a GDF9-dependent mechanism
Source: bioRxiv. 2026 Jul 23:2026.07.22.738304. Preprint. [Version 1] doi: 10.64898/2026.07.22.738304 (PMC13419792; doi:10.64898/2026.07.22.738304)
Supplement: Supplement 1 — Supplemental Figure 1: PCSK inhibition disrupts COC matrix integrity in a dose dependent manner beginning at 12 h IVM. Related to Figure 1. Brightfield images of COCs cultured in PCI at increasing concentrations or DMSO control (CTL) for 14-16 h in the EmbryoScope+™ time-lapse incubator. Supplemental Figure 2: PCSK inhibition does not affect in vitro fertilization outcomes. Related to Figure 1. Graphs showing the percentage of A) in vitro matured CTL, B) in vitro matured PCI-treated, and C) in vivo matured CTL COCs that reach various stages of embryologic development each day after in vitro fertilization. For A-C), data are represented as mean ± SEM and n=42-48 COCs per treatment group. D) Representative immunofluorescent images of blastocysts yielded from CTL or PCI-treated COCs. DNA is shown in blue, alpha-tubulin is shown in green, and rhodamine phalloidin (actin) is shown in red. Supplemental Figure 3: PCSK inhibition does not affect the morphokinetics of maturation in mouse oocytes. Related to Figure 1. A) Representative brightfield images of CTL vs. PCI-treated denuded oocytes across in vitro maturation. GV = germinal vesicle. GVBD = germinal vesicle breakdown. PBE = polarbody extrusion. White asterisks denote intact GVs and yellow asterisks denote polarbodies. B) Graphs showing time to germinal vesicle breakdown (GVBD), time to polarbody extrusion (PBE), and overall duration of meiosis in CTL vs. PCI-treated denuded oocytes. Data are represented as mean ± SEM. p > 0.05 for all comparisons by unpaired t-tests. n=24-39 COCs per treatment group. C) Graph showing maturation status of CTL or PCI-treated denuded oocytes after 16h of culture. Data are represented as mean ± SEM. p>0.05 by Welch’s t-test. n=24-39 COCs per treatment group. D) Immunofluorescent images depicting chromosomes (DAPI; blue), spindles (alpha-tubulin; green), and actin (rhodamine phalloidin; red) in CTL or PCI-treated denuded eggs. White asterisks denote spindles and yellow asterisks denote th [file media-1.pdf]

Supplemental Figure 1

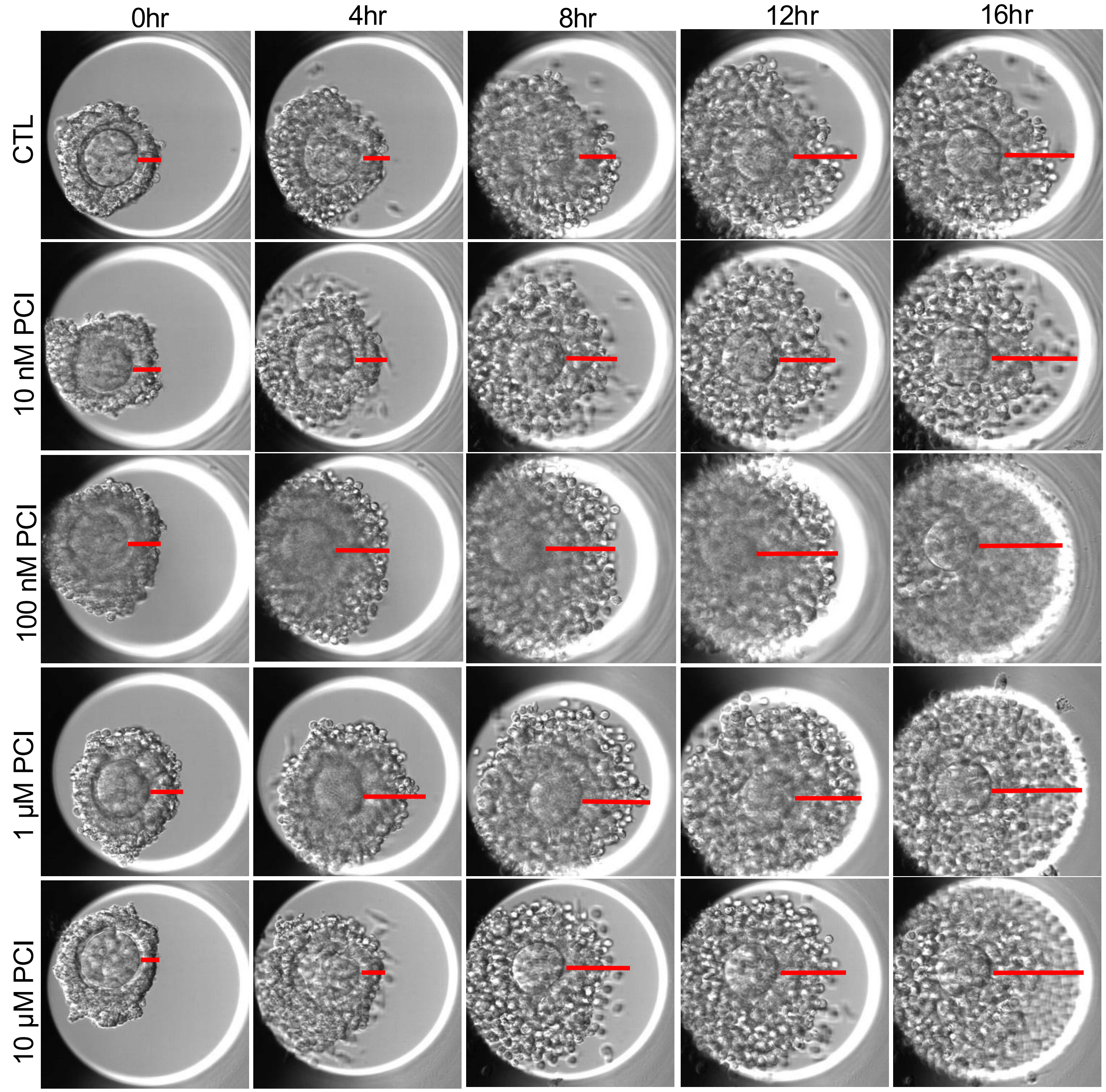

Supplemental Figure 2

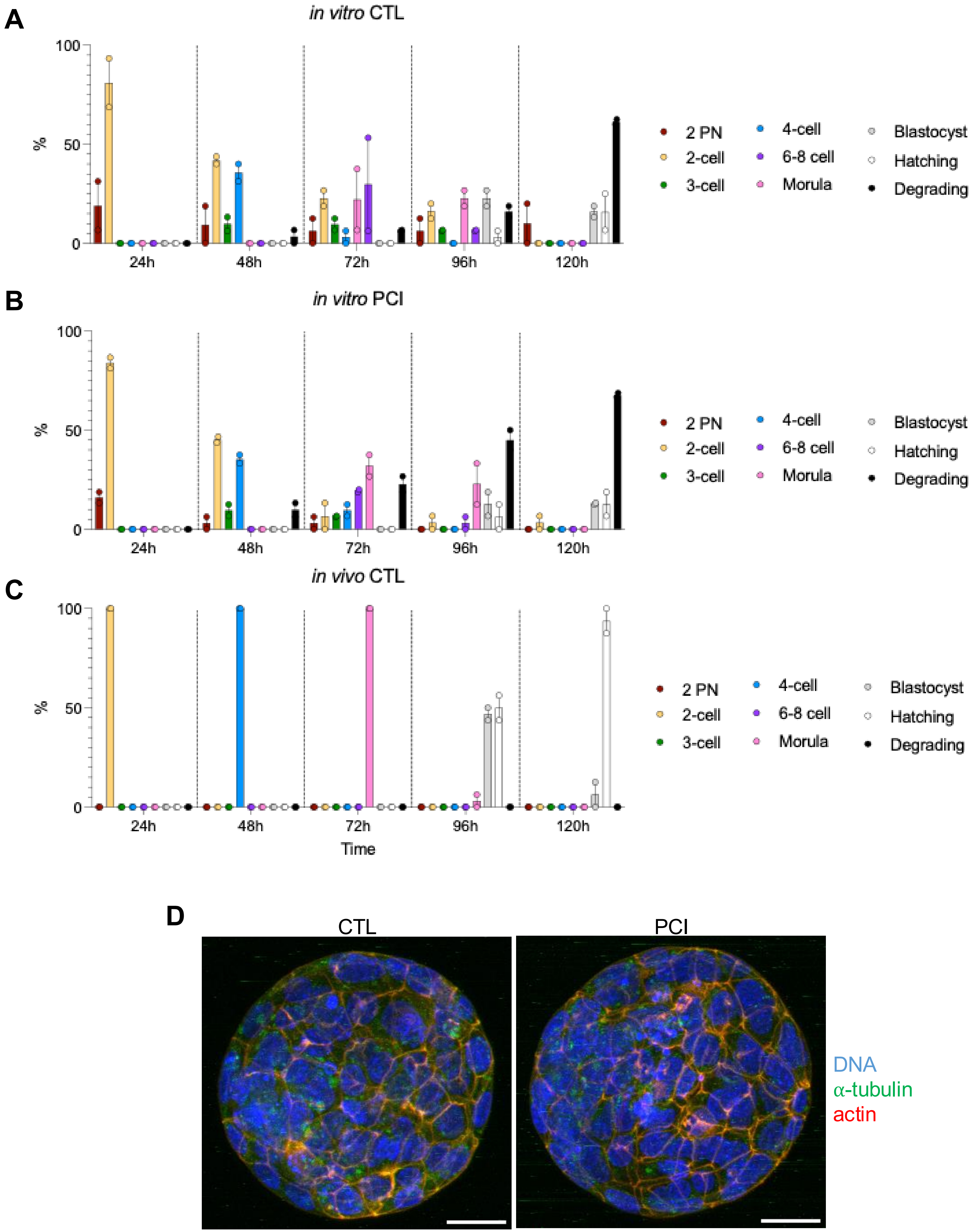

Supplemental Figure 3

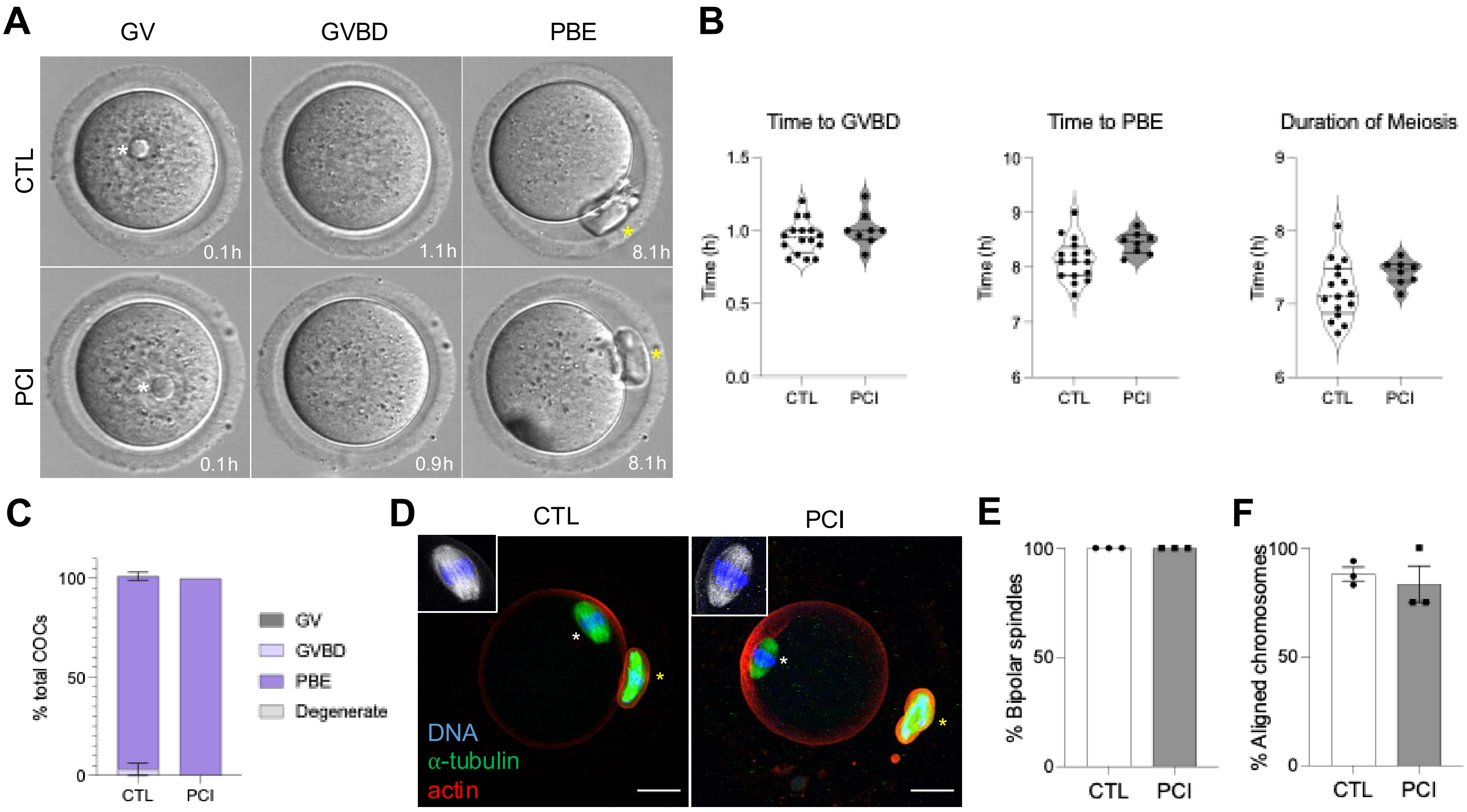

Supplemental Figure 4

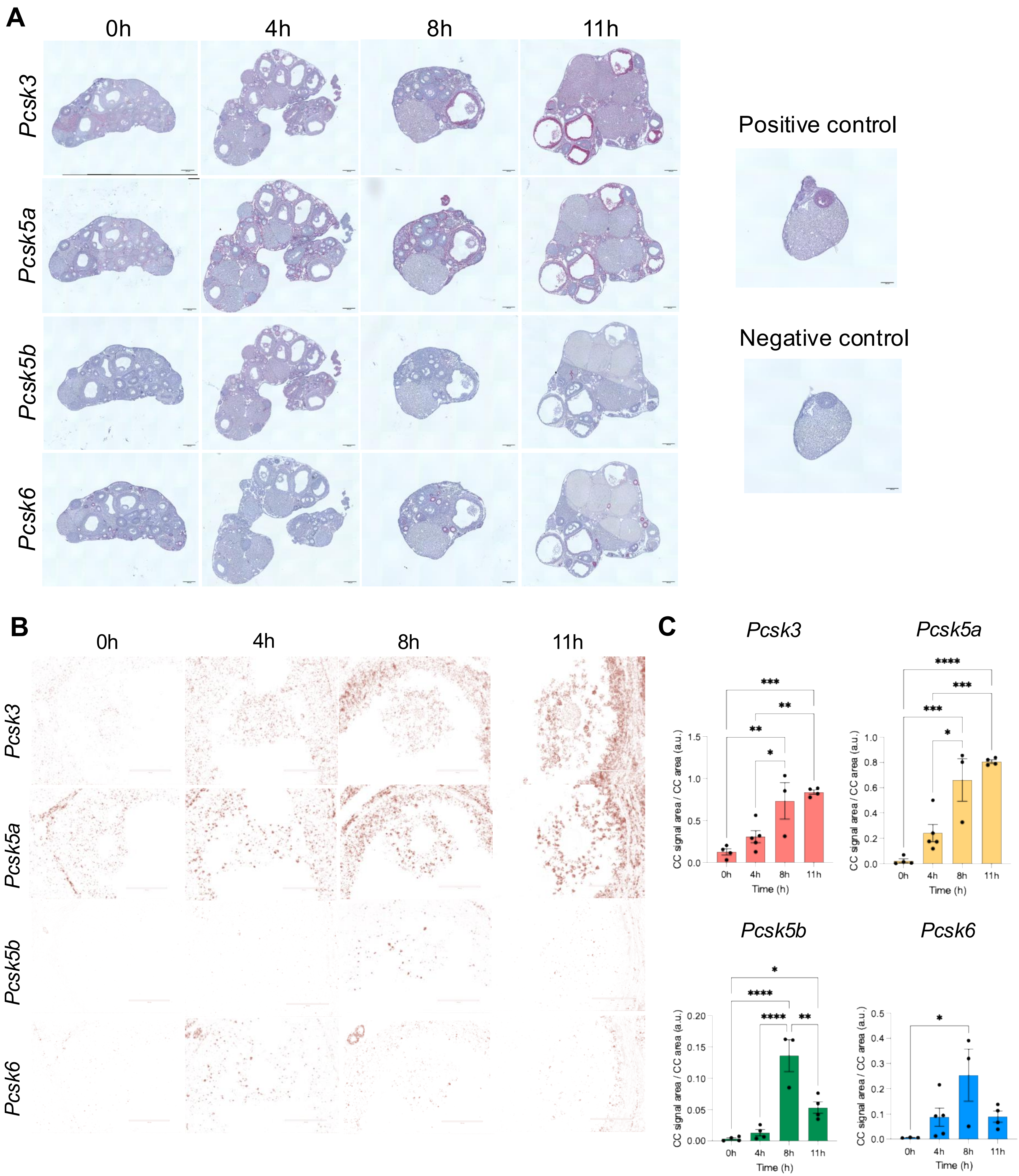

Supplemental Figure 5

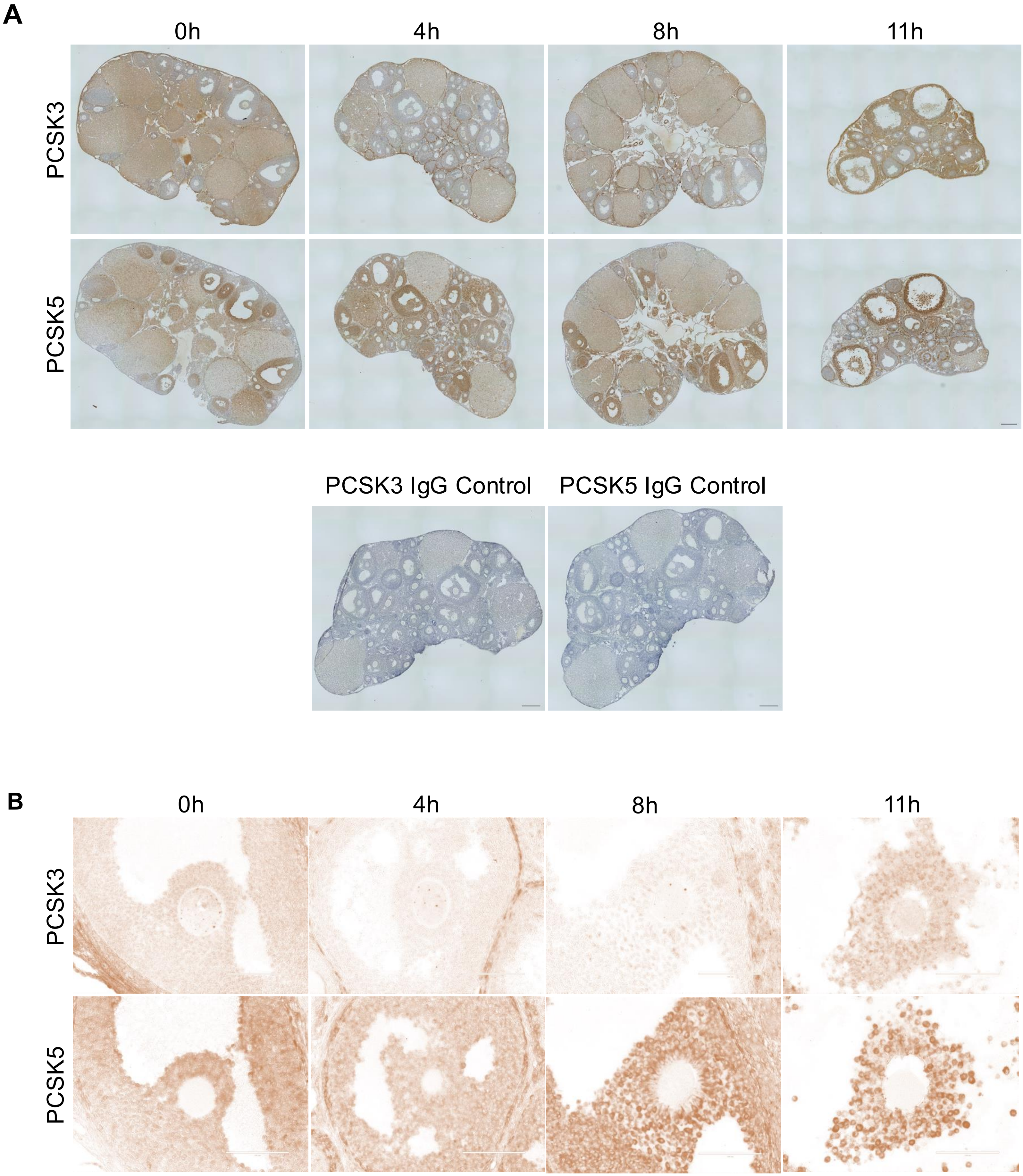

Supplemental Figure 6

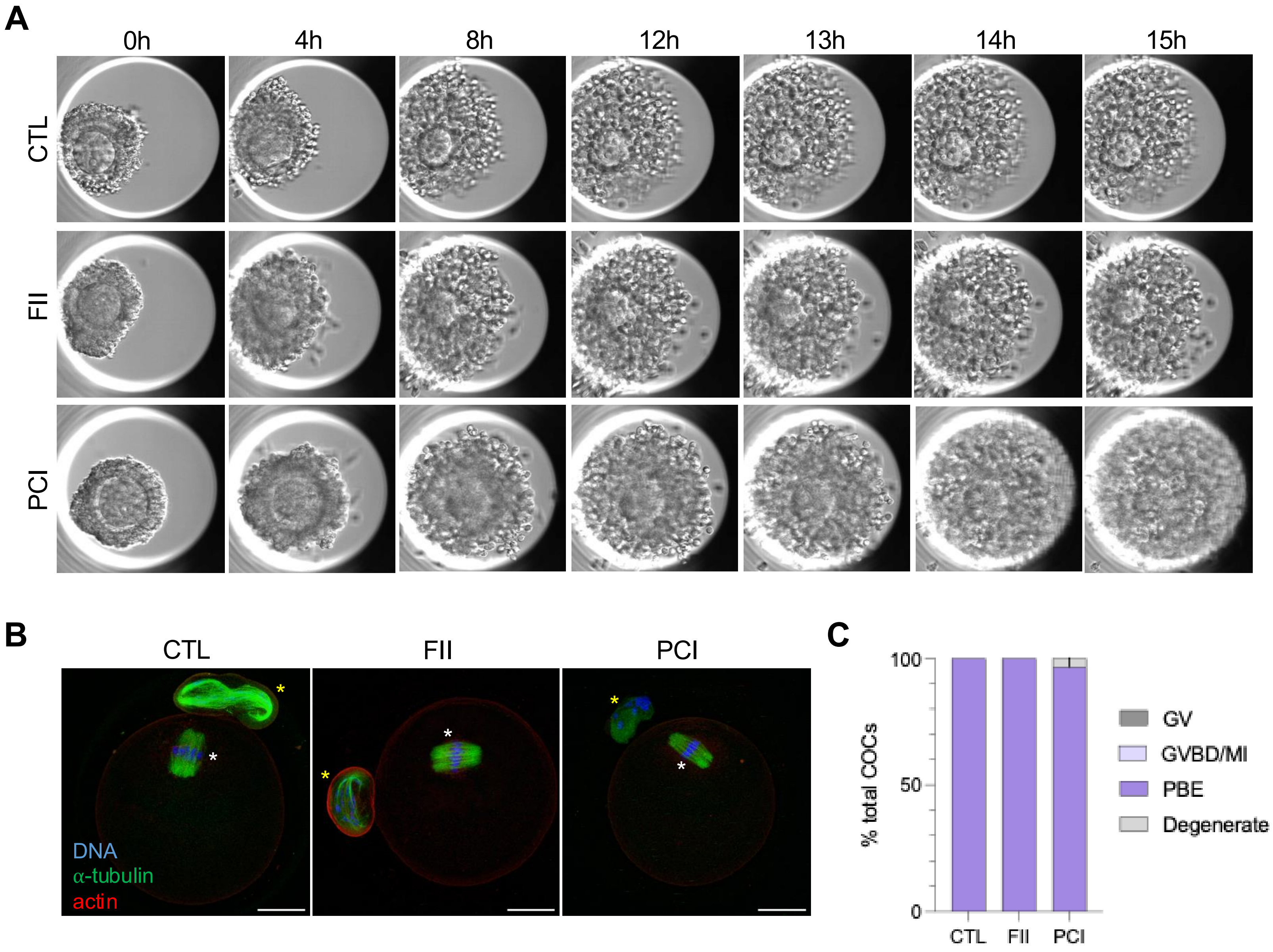

Supplemental figure 7

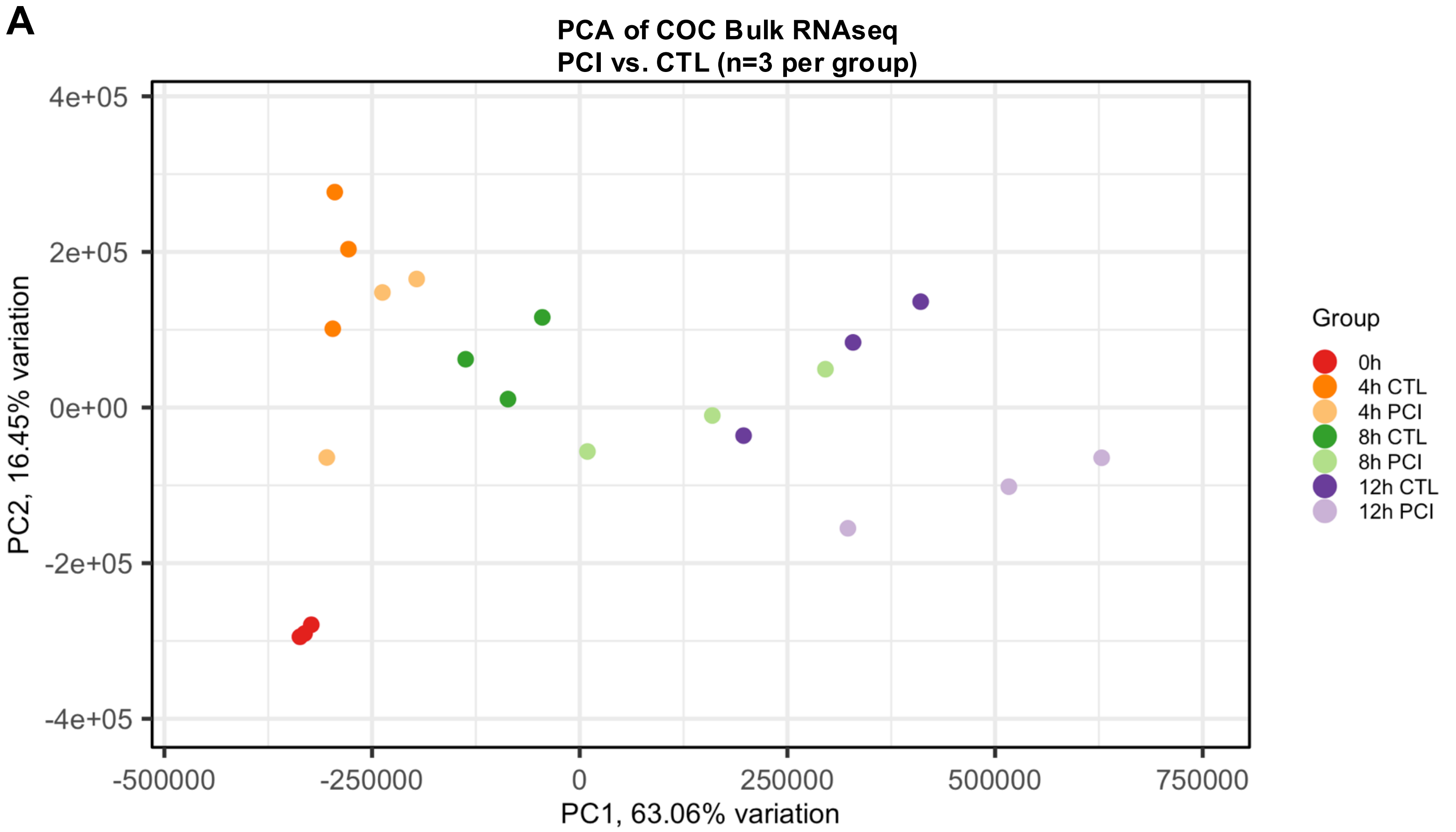

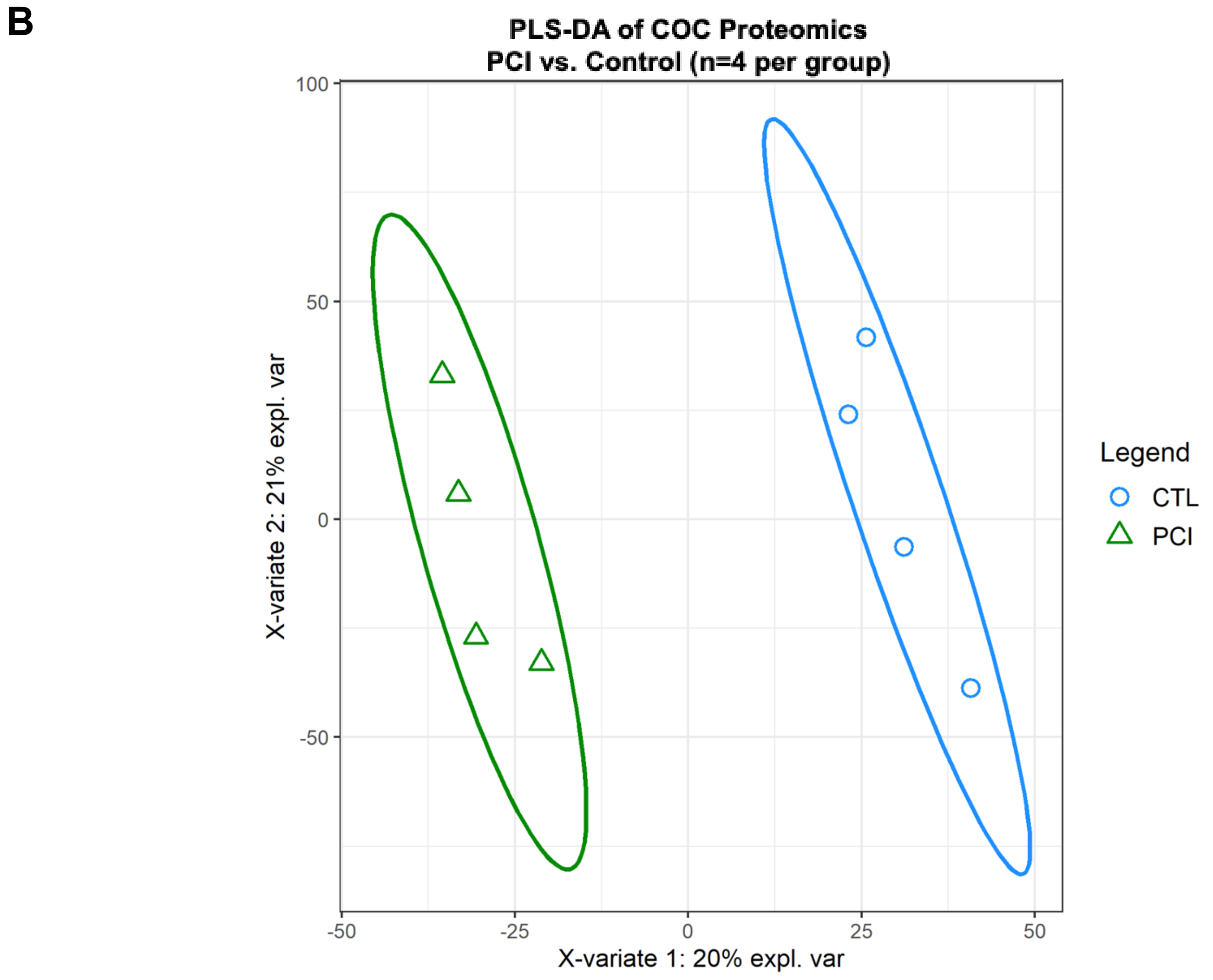

Supplemental Figure 8

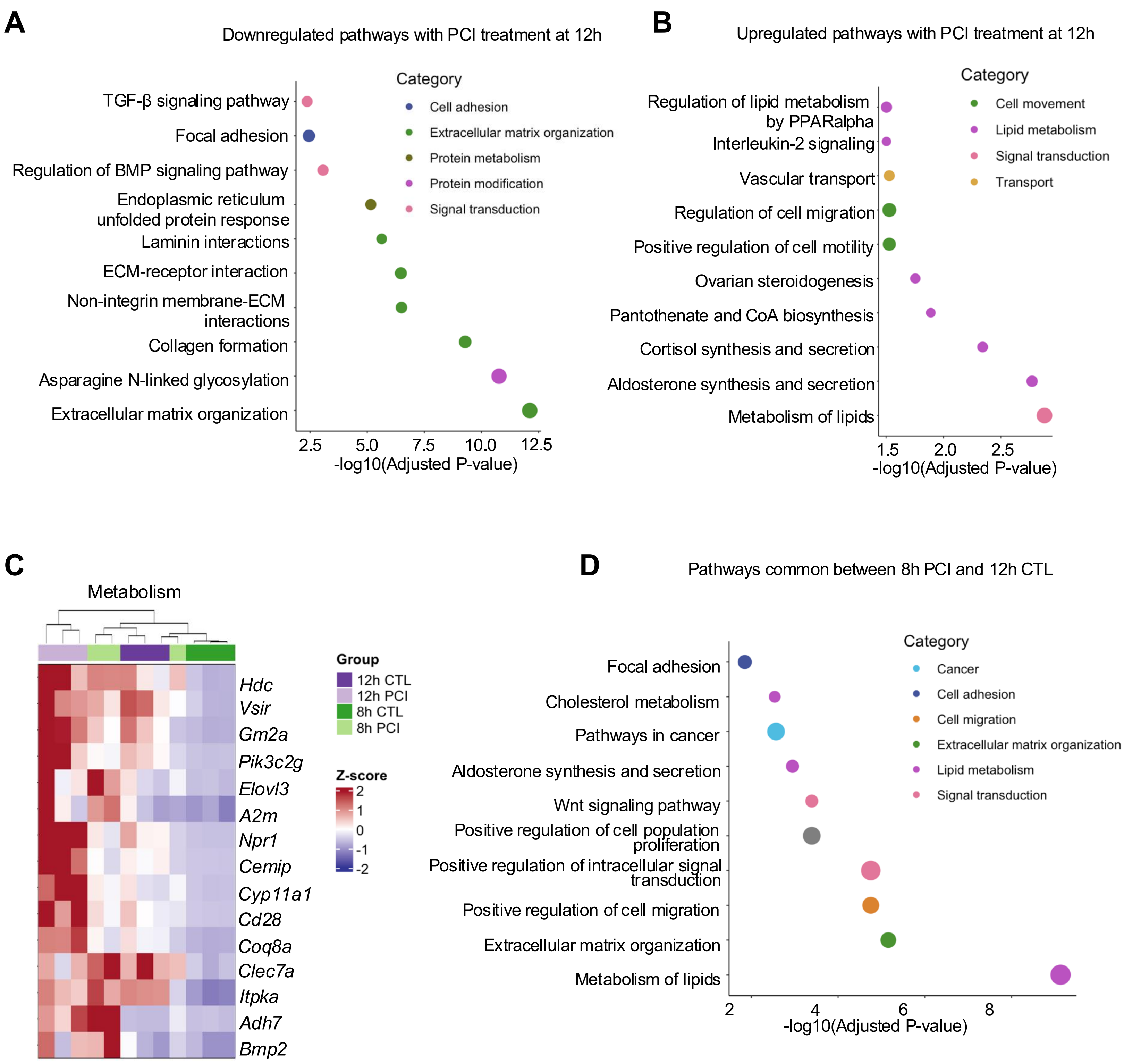

Supplemental Figure 9

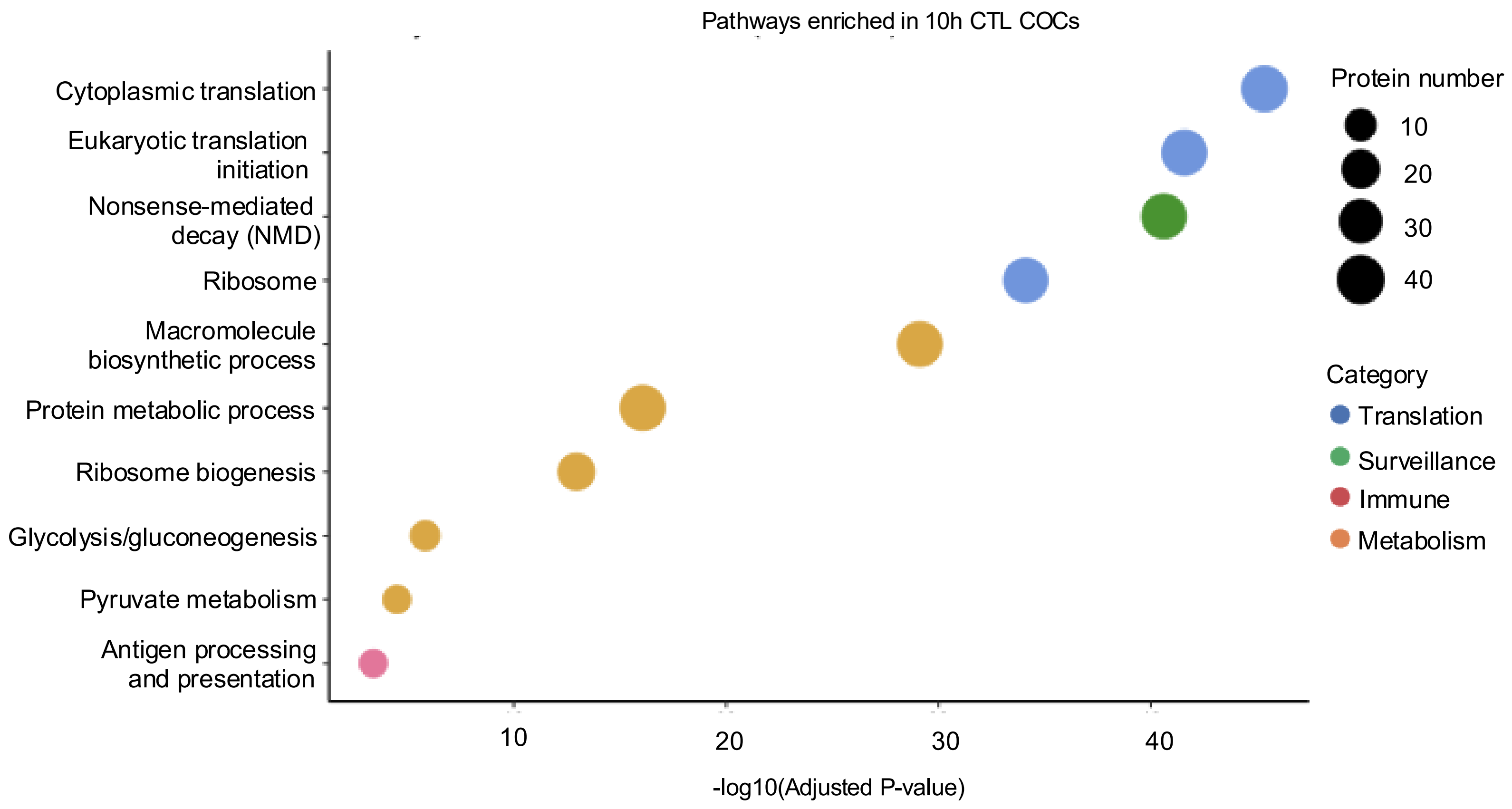

Supplemental Figure 10

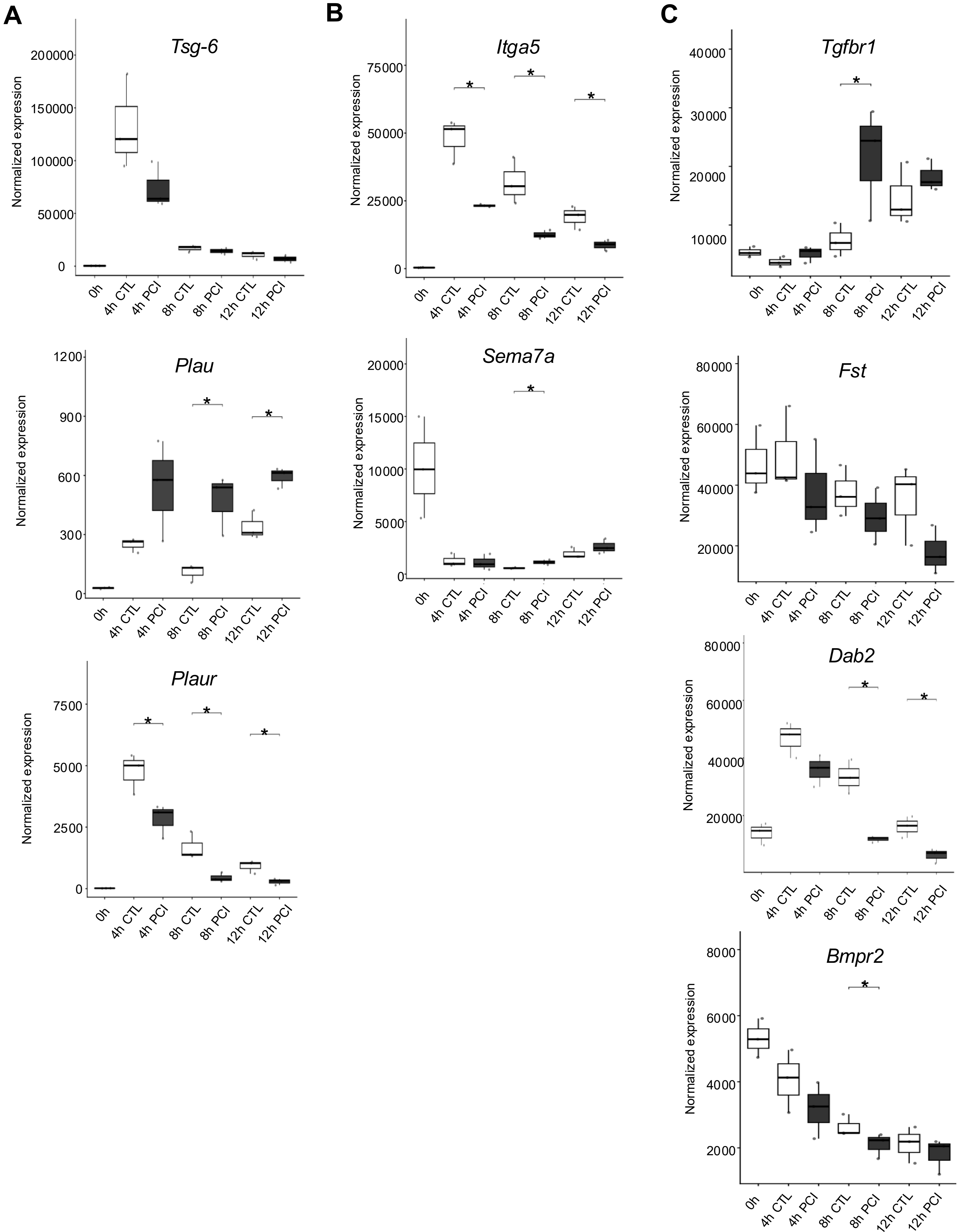

Supplemental figure 11

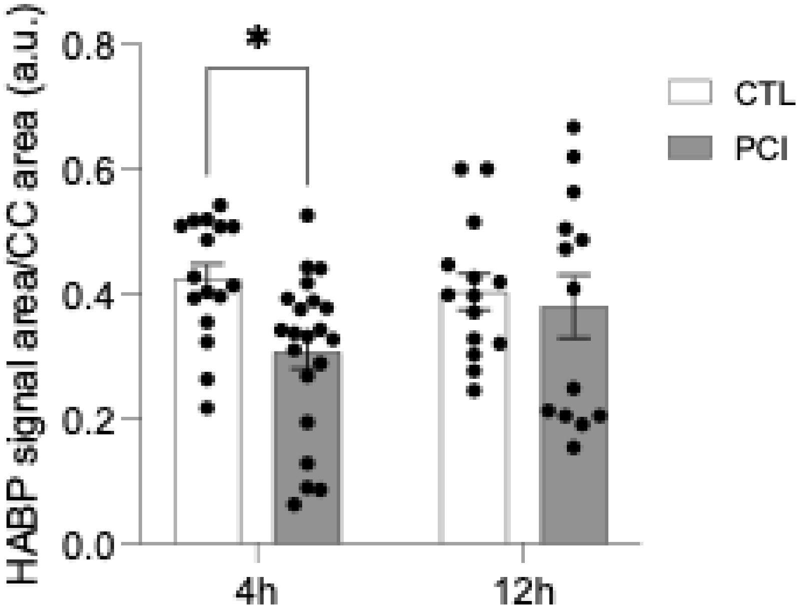

Supplemental figure 12

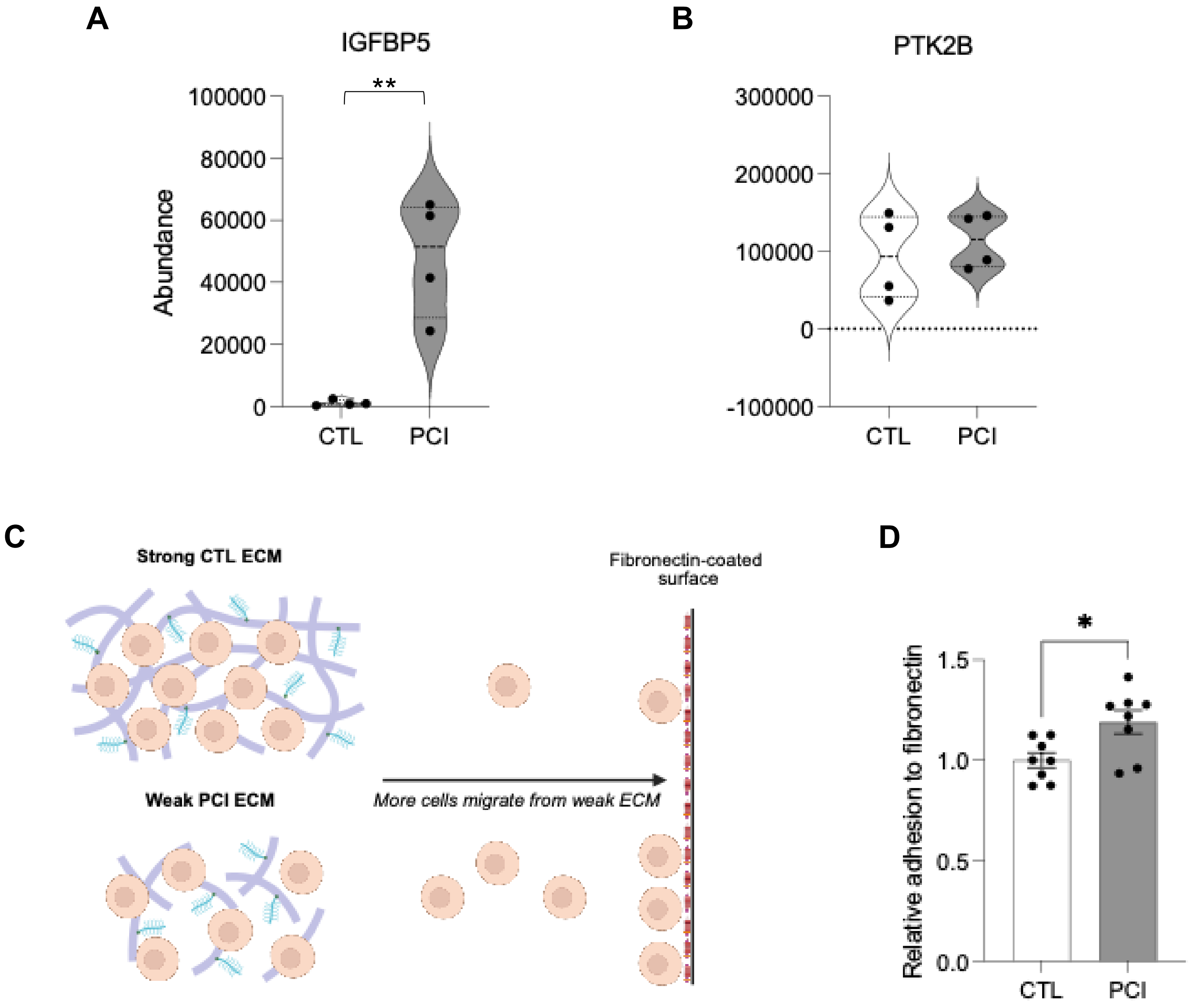

Supplemental Table 1

Category	Genes
ECM organization and cell adhesion	ITGB5; LAMB3; LAMA1; LAMA4; TNC; DMP1; LAMC2; THBS1; COL2A1; IBSP; ITGA11; COL6A3; ITGA5; VAV3; PDGFRA; JUN; CCND2; FLNC; PAK3; RAF1; FGF2; LAMA5; ITGB3; ITGA2; ITGA1; COL1A1; COL4A2; COL4A1; DAG1; SDC1; ITGAV; CD36; CD44; RASGRF1; IGF1; CAPN2; TGFB1; COL11A1; NTN4; COL18A1; COL7A1; NID1; COL16A1; PCOLCE2; COL12A1; FBLN2; SCUBE3; NCAM1; COL27A1; DST; LAMB3; MMP2; MMP12; MMP13; BMP1; LOX; P4HA2; ADAM12; ADAMTS10; FLRT2; ADAMTS19; CREB3L1; SERPINH1; HAS2; GSN; APLP1; MMP11; SMOC2; MMP14; P4HA1; P4HA3; CTSL; JAM2; P3H1; BMP4; MFAP5; MFAP2; P4HB; PLEC
Cell movement	RET; CSF1R; IGSF8; GRN; CEMIP; SEMA7A; MCTP1; PLXND1; CITED2; SEMA3B; SEMA3G; ARID4A; RORA; PTN; VSIR; EGFR; ROBO1; PPP3CA; CLEC7A; PLAU; PDGFD; GNA12; CYP1B1; FLNA; PLXNA1; PTK2B; PHACTR1; PLXNA3; CARMIL1; ANXA1; IGFBP5; FZD4; SEMA4B; INSR; CAV1; PRKCA; PPP2R3A; F3; TGFBR1; TGFBR3; BMP2; SFRP2; MYADM; SGK1; FERMT2; VCLCAVIN1; SCARB1; SPARC; SYDE1; CLASP2; FN1; GNPDA1; RIN2GRB7; SEMA3D; SEMA3A; CX3CL1; GLUL; PLXNA4; RDX; FRMD5; CXCL12; KIF26A; ANGPT1; WNT5A; PRKD1
Metabolism	RAMP2; CALCOCO1; RAMP3; DDX3X; TFRC; CITED2; IRS1; HFE; NTS; AREG; VSIR; ROBO1; C1QTNF1; HMGN5; CLEC7A; MYC; UBR5; RIPK1; SLC38A2; EGR1; MEF2C; INSR; FN1; F3; TGFBR1; TGFBR3; P2RX7; SFRP4; BMP2; RGCC; SLC6A9; CD28; LIMS1; BMPR1A; NFE2L2; ABCA1; SCARB1; MYLIP; LRP1; LPL; LIPA; SOAT1; STAR; VAPA; NPC2; ANGPTL4; LDLRAP1; LDLR; NPR1; HSD3B1; ITPR2; PRKCA; ATP1B2; ATP1A1; ATP1B1; NR4A2; LIPE; NR4A1; CREB3; PLCB3; PRKD3; CYP11A1; CAMK2G; KL; RUNX2; EGFR; PTHLH; LRP6; SLC9A3R1; MMP16; GNA12; FADS2; SCP2; ELOVL5; ELOVL3; ELOVL6; HACD3; FADS1; ACAA2; PPT1; HADH; MRAP; SLC44A3; HEXB;I NPPL1; RORA; PIK3C2G; AHR; GM2A; FDXR; ME1; CYP1B1; HMGCS2; NCOA2; PCYT1A; DGAT1; ACSL4; SUMF1; ACLY; CYP2U1; PITPNM1; FDX1; PLBD1; AHRR; FAR1; SLC27A3; PLPP2; IDI1; SLC22A5; SGMS2; ACAT2; SGPL1; ALDH3B1; PSAP; SC5D; SCAP; SPTSSA; TBL1X; HAO2; OSBPL9; CYB5B; CTSA; B3GALNT1; BCHE; CHKA; OSBPL3; CAV1; MBOAT1; PTPN13; ACSF2; SGPP1; PIKFYVE; GPAM; ETNK2; LPCAT3; DHCR7; RGL1; GLA; LGMN; PANK1; SAT1; HS6ST1; NDST2; NAGLU; PDK4; ENPP1; NUDT13; CSGALNACT2; SLC6A12; HSPG2; RBP4; BTD; CEMIP; ISYNA1; MAOA; FUT11; MGST1; GMPR; IQGAP1; ADH7; PRKAR2B; HMOX1; IP6K3; PCK2; GSTM1; GOT1; IDH1; PAICS; QDPR; GCLC; G6PC3; VNN1; AKR1B10; TCN2; KYAT3; NMT2; SERINC3; HCCS; OPLAH; NAPRT; ME2; HIBCH; TPI1; ABCC5; SORD; CTPS2; PPP2R5D; ITPKB; VCAN; TST; IVD; DPYD; ITPKA; ALDH1A1; BLVRB; R; BCAT1; ASAH1; INPP1; HDC; ODC1; PPM1K; PAPSS1; UGP2; GPC4; SLC36A4; XDH; CBR3; NQO2; HS3ST1; PDP1; GNPDA1; COQ8A; PFKP; V; PCSK5; A2M; ANGPT1; RDX; WNT5A; OLFM1; DNAJA4; ID2; TLR3; MARK1; PRKCB; ITPR1; PRKD1; CACNA1H; KCNK3; VNN3; FSHR; HSD17B7; UGCG; GAL3ST1; ENTPD1; PPAT; PDE3A; PDE4B; PDE5A; AMPD3; INSIG1; NPAS2; ARNTL; ENPP6; ARNT2; GK; SRD5A1; LPIN3; TK2; GLUL; AASS; HGSNAT ;GLCE; CACNB2; CARNMT1; SHMT1; LDHB; BRIP1; SULT1E1; PRODH; HPSE; ALAS1

Supplemental Table 2

Oocyte marker	Average Log_2 Ratio	Q-value
BMP15	0.71	0.180
GDF9	0.25	0.659
ZP2	0.08	0.367
DDX4	-0.14	0.725
NPM2	0.33	0.00841
ZAR1	0.12	0.373
YBX2	0.33	0.785
DPPA3	0.04	0.407
SUB1	0.50	0.183
ZP3	0.11	0.387
NLRP5	0.25	0.00599
ZP1	0.28	0.219
KIT	0.47	0.418
